# HDL and the golden key to cancer immunity?

**DOI:** 10.18632/oncoscience.436

**Published:** 2018-06-28

**Authors:** Michael P. Plebanek, Debayan Bhaumik, C. Shad Thaxton

**Affiliations:** Northwestern University, Feinberg School of Medicine, Department of Urology, Chicago, IL, USA

**Keywords:** high-density lipoprotein, cholesterol, myeloid derived suppressor cell, scavenger receptor type B-1, ATP-binding cassette transporter

Myeloid-derived suppressor cells (MDSCs) are a heterogeneous population of innate immune cells that play critical roles in cancer [1]. MDSCs can be categorized into two populations that resemble immature forms of either monocytes or neutrophils. MDSCs are differentiated from their cellular relatives by unique functional characteristics. Namely, MDSCs suppress T cell activation and effector T cell functions. During cancer progression, MDSCs are mobilized from the bone marrow to infiltrate tumors and immune foci leading to the development of immunosuppressive microenvironments. Multifocal suppression leads to reduced efficacy of immunotherapies, like checkpoint inhibitors and cell therapies, that rely upon an active adaptive immune response to clear cancer [2]. Agents that inhibit MDSCs may prevent cancer, reduce metastasis, and increase the efficacy of therapies aimed at harnessing adaptive immunity to eradicate advanced disease.

There is tremendous interest in identifying factors that control MDSCs. But first, the role of cholesterol in myeloid cells deserves attention. In 2010, Yvan-Charvet et al discovered that knocking out ATP-binding cassette transporters, ABCA1 and ABCG1, increased proliferation of myeloid cells [3]. ABCA1 and ABCG1 are responsible for effluxing cellular cholesterol to apolipoprotein A-I (apoA-I) containing high-density lipoproteins (HDL). ApoA-I is the main protein constituent of HDLs. Cholesterol efflux to HDLs, particularly through ABCA1, promotes the formation of “mature”, cholesterol-rich, spherical HDLs that can deliver their payload of cholesterol to cells by binding scavenger receptor type B1 (SR-B1). Yvan-Charvet et al reported that administration of exogenous HDL to mice lacking ABCA1 and ABCG1 reduced the myeloproliferative phenotype to baseline suggesting that HDLs suppress myeloid cell proliferation independently of these receptors. These data implicate other receptors for HDL, particularly SR-B1, in the control of myeloid proliferation. At the time, connections between HDL, cholesterol, and myeloid cells during cancer progression were not fully appreciated.

Studies began to link these concepts as Zamanian-Daryoush et al demonstrated in mouse models of cancer that mice lacking apoA-I had increased tumor growth and metastasis [4]. Additionally, high-dose infusion of apoA-I reduced metastasis and tumor burden. Although, the mechanism through which apoA-I functioned was not identified, the authors found that there were reduced MDSCs in the tumor microenvironment after apoA-I treatment. Based on the HDL-mediated reduction of myeloid cell hyperproliferation discussed earlier, we postulated that the anti-cancer effect of apoA-I infusion was due to the *in vivo* maturation of a portion of the apoA-I to cholesterol-rich HDL which subsequently bound SR-B1 to inhibit MDSCs.

Exploring this, we recently provided a missing link to understanding MDSC function and the role of SR-B1. The findings were enabled by our synthetic HDL nanoparticle (HDL NP) that binds specifically and tightly to SR-B1. The surface of HDL NPs is endowed with apoA-I and phospholipids to mimic cholesterol-rich HDLs and enables recognition by SR-B1. However, HDL NP critically differs from the cholesterol-rich HDL counterpart due to its interior content. HDL NPs are synthesized using a 5 nm diameter gold nanoparticle as a core scaffold that imparts a static spherical conformation that appears to be a mature cholesterol-rich HDL when, in fact, it is cholesterol poor. In mouse models of melanoma and lung cancer, our data demonstrated that HDL NP infusion potently inhibits the activity of MDSCs resulting in a robust T cell-mediated immune response to cancer [5]. HDL NPs, at low dose, slowed primary tumor growth, drastically reduced metastasis, and significantly increased overall survival. More specifically, HDL NPs reduced MDSCs, increased CD8^+^ T cells and reduced T regulatory cells in the metastatic microenvironment. Importantly, therapeutic effect was mediated specifically by SR-B1 expression, as HDL NPs were unable to trigger these responses in mice with SR-B1-null myeloid cells.

It is important to rationalize our data in the context of the other published work and understand myeloid cell function in the context of HDL cholesterol. Additionally, previous works studied the impact of HDLs in cancer by knocking out components of HDL-mediated cholesterol transport pathways. Far less is known of the effects and mechanisms of HDLs in wild-type models. In our case, exquisite targeting of SR-B1 by HDL NPs has a profound impact in cancer without the need for genetic manipulation. To rationalize these effects, we must understand the nature of SR-B1 and the state of mature HDLs in each system. Cholesterol-rich HDL binds with a higher affinity to SR-B1 than immature HDLs. Mature HDLs bound to SR-B1 deliver cholesterol, reduce in size, and are then released from the receptor [6]. As discussed earlier, HDL NPs are cholesterol poor but have a static mature conformation, allowing them to remain bound to SR-B1 and outcompete natural HDL. Our data support that potent HDL NP binding of SR-B1 results in inhibition of MDSC function. In the case of ABC transporter knockout mice there is accumulation of immature lipid-poor discoidal HDLs in the peripheral blood [7]. This means there are few mature HDLs available to bind SR-B1 and suppress myeloid cell proliferation. Considering the apoA-I-null model, there are no mature HDLs to bind SR-B1 resulting in increased MDSCs. Therefore, collective data support that HDLs and HDL NPs target SR-B1 to inhibit MDSC function.

**Figure 1:**
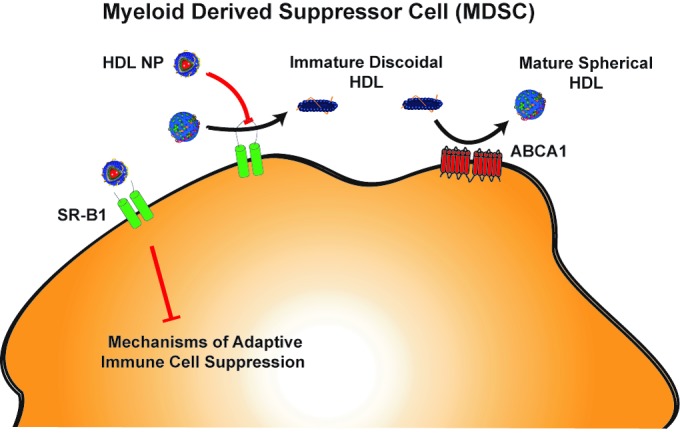
High-density lipoprotein (HDL) and HDL nanoparticle (HDL NP) interactions with myeloid derived suppressor cell (MDSC)

In humans, studies demonstrate that serum levels of apoA-I and HDLs correlate with reduced cancer incidence, metastasis and cancer-associated mortality [8]. This may be due to HDL-mediated inhibition of MDSCs through SR-B1 and enhanced anti-tumor immunity. Therefore, HDL and HDL NP, in particular, may be an extremely effective preventive, anti-metastatic, and targeted therapy for advanced cancer especially in combination with other drugs, including ones that modulate the immune system.
